# Bromine Transformation during Catalytic Pyrolysis of Waste Electronic Circuit Boards (WECBs) in an Auger Reactor over the Dual-Catalyst HZSM-5/CaO

**DOI:** 10.1021/acsomega.5c08152

**Published:** 2025-11-06

**Authors:** Samina Gulshan, Hoda Shafaghat, André Selander, Hanmin Yang, Panagiotis Evangelopoulos, Pär G. Jönsson, Weihong Yang

**Affiliations:** † Department of Materials Science and Engineering, 7655 KTH Royal Institute of Technology, Brinellvägen 23, SE-114 28 Stockholm, Sweden; ‡ Division of Bioeconomy and Health, Department of Biorefinery and Energy, 388792 RISE Research Institutes of Sweden AB, SE-941 28 Piteå, Sweden; § Paper and Forest Product Manufacturing, SCA Munksund AB, SE-941 87 Piteå, Sweden

## Abstract

Effective bromine mitigation is a critical challenge in the sustainable recycling of electronic waste, where the uncontrolled release of brominated species compromises both environmental safety and product quality. This study unveils a novel synergistic transformation pathway of bromine (Br) during ex situ dual-catalyst pyrolysis of waste electronic circuit boards (WECBs). Experiments were conducted in a continuous auger reactor integrated with a fixed-bed catalytic unit employing a dual (HZSM-5/CaO) catalyst system. By tuning the weight hour space velocity WHSV from 0.6 to 1.0 h^–1^, the catalytic process not only doubled the gas yield from 2.7 to 6.5 wt % but also selectively suppressed liquid formation from 18.0 to 12.5 wt %, while driving deeper deoxygenation and aromatic hydrocarbon enrichment. At lower WHSV, intensified secondary reactions promoted the generation of lighter aromatics and also accelerated coke deposition, highlighting the need for WHSV optimization. Mechanistic insights reveal that brominated phenols and aromatic hydrocarbons dominate the primary volatile fraction, where Br^+^ radicals undergo dual pathways: recombination with H^+^ and small fragments forming HBr/CH_3_Br, or neutralization by CaO to yield stable CaBr_2_. Importantly, 44 wt % of total bromine was retained in the solid residue as CaBr_2_, drastically lowering bromine content in pyrolysis oils. The dual-catalyst strategy thus enables simultaneous Br-fixation, hydrocarbon upgrading, and catalyst regeneration, drastically reducing bromine in pyrolysis oils. These findings provide a scalable, mechanistically guided route for the valorization of cleaner electronic waste, coupling environmental protection with high-value fuel production.

## Graphical abstract

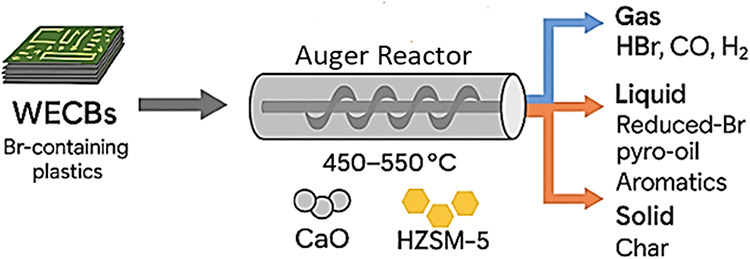

## 1 Introduction

In recent decades, advancements in information technology have sparked a revolution, leading to a global surge in the demand for electrical and electronic equipment. Simultaneously, the lifespan of these devices has been decreasing. As a result, the volume of electrical and electronic equipment waste (WEEE) has increased significantly, and according to recent projections, it is expected to grow exponentially.^1^

Printed circuit boards (PCBs) are essential components in electronic devices, accounting for approximately 3–5% of the total mass of electronic waste.^2^ PCBs are among the most complex and valuable components of WEEE. In 2019, global waste printed/electronic circuit board (WPCB) generation reached 3.2 million tons, anticipated to exceed 4.5 million tons by 2030.^3^ PCBs are composed of metals (e.g., gold, silver, platinum, palladium), nonmetals (e.g., plastics), and nonmetallic materials (NMMs) such as glass fibers, epoxy resins, and brominated flame retardants (BFRs).^4^ By 2035, they are expected to generate 5.7 million tons of resin waste, 2.311 million tons of copper, and 333.5 tons of gold.^5^

Nonmetallic materials in PCBs contain several harmful substances, including brominated flame retardants (BFRs), which comprise 5–20% of their composition. This dual nature of being a valuable resource and a hazardous pollutant makes PCBs challenging to dispose.^6^ Traditional methods, such as incineration and landfilling, have led to significant environmental issues. In landfills, toxic BFRs can leach into soil and water, causing environmental contamination, while incineration releases bromine-containing pollutants, such as polybrominated dibenzofurans, into the atmosphere, resulting in secondary pollution.^7^ Consequently, safe and environmentally friendly disposal methods, including debromination, are essential for handling waste printed/electronic circuit boards effectively.

Pyrolysis is a promising technology for treating and recycling WEEE due to its fast reaction time and high process efficiency.^8,9^ Pyrolysis converts waste into valuable products such as oil, syngas, and solid residue, which can be utilized in various energy applications, promoting the efficient recycling of materials and chemicals. Liu et al. investigated the microwave copyrolysis of industrial sludge and waste biomass, demonstrating that the addition of waste biomass improved gas and bio-oil yields while reducing the production of biochar. The process produced bio-oil with low oxygenates and fewer nitrogen compounds, while enhancing biochar quality and stabilizing heavy metals.^10^ Evangelopoulos et al. performed research focusing on the reaction pathways for thermal decomposition of PCBs fraction (thermogravimetric analysis, TGA) under N_2_ and steam atmosphere for different heating rates and for analytical pyrolysis (Py-gas chromatography-mass spectrometry, Py-GC/MS).^11,12^ These researches revealed that the thermal degradation of PCBs occurs in two distinct stages: an initial phase between 250 and 370 °C characterized by rapid decomposition and a subsequent phase at higher temperatures marked by slower mass loss.^11^ Analytical pyrolysis identified several pyrolysis products, with phenol being the most abundant.^12^ This investigation suggests that recovering the monomer through feedstock recycling could be a promising approach. However, the high bromine content in PCBs leads to the generation of brominated pollutants during heating treatment, such as CH_3_Br, HBr, SbBr_3_, 2,6-bromophenol, and methyl bromide. These substances can cause equipment corrosion and present significant environmental hazards.^13^

Consequently, the direct use of pyrolysis-derived products, such as oil and gas, as feedstocks or for chemical recycling remains a significant challenge. A thorough understanding of bromine’s migration during the pyrolysis of waste printed/electronic circuit boards is crucial for effectively reducing emissions. Ortuño et al. conducted a study on pollutant emissions during the pyrolysis and combustion of WPCBs, discovering that hydrogen bromide (HBr) is the primary gaseous product released during the decomposition of printed circuit boards, with its formation increasing as the temperature rises.^14^ Zhu et al. investigated bromine removal for waste-printed circuit boards under low-temperature vacuum pyrolysis. They concluded that at the temperature of 300 °C, most of the bromine in resin particles was transferred to the oil, and this not only reduced energy consumption but also reduced the generation of toxins compared to high-temperature vacuum pyrolysis.^13^

Employing efficient catalysts or additives is beneficial for removing bromine from pyrolysis products. Several studies have used various catalysts such as metal and metal oxides,^15^ acids and alkalis,^16^ carbon-based catalysts and zeolites^17^ for the catalytic pyrolysis of printed circuit boards to eliminate the bromine contamination. Zhao et al.^18^ performed a thermogravimetric analysis coupled with FTIR to analytically study the catalytic effect of MgO, CaO, and HZSM-5 on the pyrolysis of WPCBs. The results revealed that the primary products from the thermal decomposition of brominated epoxy resin are phenol and aromatic hydrocarbons. Moreover, using ZSM-5 significantly increased the mass loss rate compared to other catalysts and broadened the main decomposition temperature range from 340–375 to 340–480 °C. Wang et al. reviewed catalytic pyrolysis for the debromination of waste printed circuit boards (WPCBs) and concluded that it is a promising strategy, with transition-metal catalysts, particularly bimetallic systems, offering high efficiency and low energy demand. They further emphasized that optimal operating parameters, especially temperature and catalyst type, are crucial for effective bromine removal and the generation of valuable products.^19^ In another study, Wang et al. investigated the catalytic pyrolysis of waste printed circuit boards (WPCBs) using a Cu/Fe bimetal catalyst and demonstrated the synergistic effect between copper and iron. They found that Cu facilitated the conversion of organic bromine species (bromophenol and bromomethane) into inorganic forms (HBr and Br_2_), which were subsequently fixed by Fe in the residue.^20^ Gao et al.^21^ found a reduction in bromide content in the pyrolysis oil by applying a CaCO_3_ coating to the WPCB, obtaining a pyrolysis oil with a phenol content as high as 89.57 wt %. In another study, Gao et al.^22^ concluded that Ca­(OH)_2_/Cu additive facilitated the conversion of organobromine compounds into Br_2_/HBr by weakening and cracking the C–Br bond. Zhang et al.^23^ investigated the product characteristics and bromine migration of WPCBs during catalytic pyrolysis using HZSM-5 and kaolin catalysts in a microwave-assisted auger reactor. This study concluded that the addition of HZSM-5 and kaolin increased the relative percentage of monocyclic aromatic hydrocarbons except for phenols and increased the Br-fixation efficiency in pyrolysis residue.

Most studies on the pyrolysis or catalytic pyrolysis of waste printed/electronic circuit boards (WPCBs) have been limited to lab-scale batch reactors with in situ configurations, providing only preliminary insights into product distribution. In contrast, little attention has been given to the fundamental mechanisms of bromine migration, particularly the immobilization of Br in liquid and solid fractions under ex situ catalytic conditions. Moreover, research on dual-catalyst systems in a pilot-scale, continuously operated pyrolysis reactor remains unexplored, leaving a critical gap between laboratory feasibility and industrial application. Therefore, this study investigates the pyrolysis of waste electronic circuit board (WECBs) with and without a catalyst in a continuously operated auger reactor, followed by a fixed catalysis system applying a dual mix mode of the HZSM-5/CaO catalyst in a 1:1 wt % ratio. A comprehensive analysis of the formation of lighter hydrocarbons and fixation of Br-containing substances in solid residue and oil was performed. In addition, the decarbonization of the studied process was also examined using CaO catalysts in combination with zeolite. To complement this, a lab-scale batch thermal and catalytic pyrolysis was conducted using only a zeolite catalyst in an ex situ configuration.

## 2 Materials and Methods

### 2.1 Waste Electronic Circuit Board Material

The feedstock used in this study was a printed circuit board named waste electronic circuit board (WECB) in the form of pellets. This sample was provided by Boliden Rönnskär in Sweden and shredded into a particle size of ≤6 mm, as shown in Figure 1. The feedstock was extensively mixed to have a homogeneous sampling before experiments. Ultimate, proximate, and element analyses were conducted by Eurofins Environmental Testing Sweden AB using standard methods SS-EN ISO 21663:2020, SS-EN 15403:2011, and SFS-EN ISO 16968:2015-2016 as presented in Table 1. The primary elements identified in the element analysis include copper (Cu), aluminum (Al), calcium (Ca), silicon (Si), tin (Sn), and bromine (Br). Cu is the most abundant element in WECBs, mainly used in conductive tracks and connectors. The high proportion of Si is primarily attributed to the presence of glass fiber substrates in the WECBs. The elements Br, P, and Sb likely originate from brominated flame retardants in WPCBs.^24^

**Figure 1:**
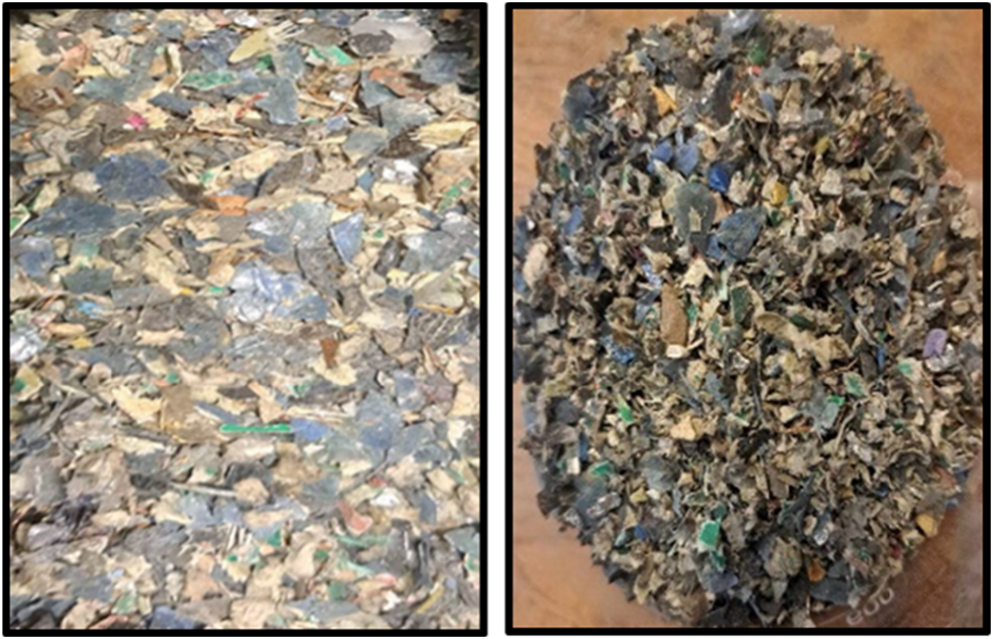
Feedstock (WECB).

**Table 1:** Proximate, Ultimate, and Elemental Analyses of WECB (O* is Calculated by Difference)

proximate analysis (wt %, db)		
	result	Unc %
moisture content	0.7	10
ash content	60.97	10
volatile matter	40.9	5
heating value (MJ/kg)		
HHV	14.06	
LHV	13.17	5

ultimate analysis (wt %, db)		
	result	Unc %
C	24.2	5
H	2.2	10
N	0.61	10
Cl	0.91	25
S	0.07	10
O*	11.2	

elemental analysis (mg/kg, dry basis)	
bromine	15,000
aluminum	61,000
antimony	3900
arsenic	18
barium	3400
beryllium	<0.40
lead	5300
boron	2500
phosphorus	570
iron	3000
cadmium	1.1
calcium	41,000
potassium	350
silicone	34,000
cobalt	13
copper	200,000
chromium	94
mercury	0.09
magnesium	4000
manganese	140
molybdenum	8.5
sodium	1000
nickel	1400
tin	47,000
titanium	920
vanadium	13
zinc	7700

### 2.2 Catalyst Preparation

Commercial zeolite HZSM-5 with SiO_2_/Al_2_O_3_ ratio of 38:1 was supplied by ACS Materials LLC. Calcium oxide (CaO, 99.99% trace metals basis) was purchased from Merck. HZSM-5 and CaO were mixed in a weight ratio of 1 in this study, configured as dual catalysts. This ratio was identified as optimal for achieving high conversion of pyrolysis vapors into aromatic hydrocarbons, as demonstrated in the author’s previous work.^25^

Before tests, the HZSM-5 catalyst was calcined in a muffle furnace at 550 °C for 6 h. CaO was prepared by calcination at 800 °C for 4 h. After the calcination, these two catalysts were mixed with particle sizes ranging between 3 and 10 mm. This particle size was chosen to fully occupy the bed volume without creating a pressure drop and disturbing the flow of volatile products. Characteristics of catalysts are presented in Table 2.

**Table 2:** Characteristics of the Catalysts

catalyst	HZSM-5	CaO
shape	pellets	pellets
dimension	3–10 mm	3–10 mm
pore volume	≥0.25 cm^3^/g	0.13 cm^3^/g
bulk density	∼0.72 kg/L	800–1200 kg/m^3^
specific surface area	≥250 m^2^/g	13.4 m^2^/g

### 2.3 Batch Pyrolysis

Thermal and catalytic pyrolysis of WECB were performed using a lab-scale batch pyrolyzer as a complementary run for the continuous experiments. Thermal pyrolysis was carried out in an electrically heated reactor to 500 °C, with a controlled heating rate of approximately 5 °C/min. An inert nitrogen (N_2_) carrier gas was used at a flow rate of 2.5 L/min to transport the pyrolysis vapors through the system. In the catalytic pyrolysis of WECB, the pyrolysis vapors were carried by N_2_ through an ex situ catalytic bed packed with HZSM-5 pellets maintained at 450 °C. These parameters were selected based on the author's previous study.^26^

### 2.4 Continuously Operated Auger Reactor

The experimental setup used in this study is presented in Figure 2. The continuous system consists of an auger pyrolysis reactor, a vertical fixed-bed catalytic reactor, a cooling system, and an online gas analysis system.

**Figure 2:**
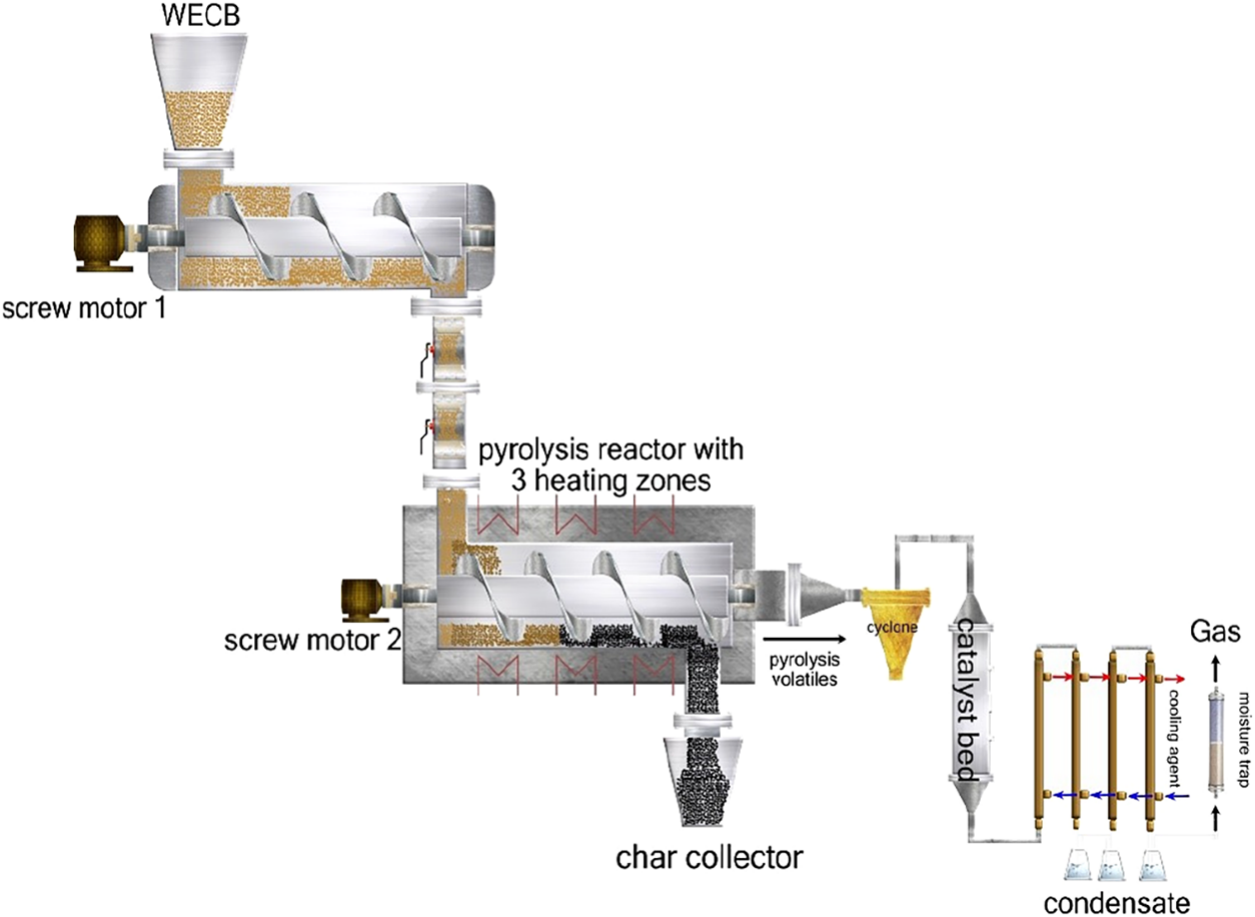
Experimental apparatus of pyrolysis of WECB in an auger reactor.

The auger reactor used in this study consists of two screw feeders. The primary screw feeder provides a constant and continuous feeding of the feedstock. The optimal feeding rate was determined for each experiment by calibrating the feeding rate against the rotational speed of the screw. This calibration curve can be seen in Figure S1. It is essential for this investigation that the WECB fraction is homogeneous in terms of particle size to ensure a constant feeding rate. The second feeding system is located inside the reactor, with a spiral outer diameter of 54 mm and a total length of 1200 mm. Two pneumatic valves are placed between the screw feeders 1 and 2 to provide continuous material flow. The constant flow of nitrogen is introduced between these two valves to empty the air that enters the system. Furthermore, another nitrogen flow is introduced to the reactor to control the vapor flow and retention time. The constant flow rate of nitrogen within the reactor was 1 L/min. The auger reactor consists of three heating zones along the reactor, which can be set to desired temperatures independently to ensure a homogeneous temperature profile. The heating zone closer to the inlet of the reactor was set to 300 °C for all experiments to avoid immediate melting of the plastic material in the feedstock, which might result in the inlet blockage. Heating zones 2 and 3 were set to 500 °C and the temperature of the fixed-bed catalytic reactor was set to 450 °C according to our previous study.^26^

Before the start of each test, the vertical fixed-bed reactor was filled with the dual-catalyst HZSM-5/CaO. Detailed test cases and corresponding conditions are summarized in Table 3. The temperature of the catalytic bed was set to 450 °C for all the experiments. In this study, different WHSV was investigated by changing the feeding rate of the feedstock while keeping the catalyst load amount constant. The maximum catalyst loading volume was 400 cm^3^, and the quantities of both catalysts were determined based on their densities and the specified loading volume. WHSV was set to 0.6 and 1 for the 1.6 and 2.5 g/min feeding rate. A noncatalytic run was also performed as a reference case (without a catalyst). The whole experiment lasted for 3 h.

**Table 3:** Experimental Plan

	HZSM-5/CaO (M)	WHSV (h^–1^)	feeding rate (g/min)	*T*_pyr_ °C	*T*_cat_ °C
1	non-cat		1.6	500	450
2	1:1	0.6	1.6	500	450
3	1:1	1	2.5	500	450

At the end of the pyrolysis process, three main products, solid residue, oil, and gas, were collected and analyzed. The solid residue was collected at the bottom of the screw reactor. The oil yield was obtained by weighing the washing bottles and connecting pipes at the bottom of the second reactor before and after each run, thereby accounting for the condensed liquid products. The amount of noncondensable gases was calculated based on their composition and flow rates, measured using a Micro Gas Chromatograph (Micro GC). Coke formation was assessed by comparing the weight of the catalyst before and after pyrolysis.

### 2.5 Analysis Methods

#### 2.5.1 Liquid Analysis

The liquid product obtained from pyrolysis/catalytic pyrolysis was separated into two distinct phases: an aqueous phase and an organic phase, and each phase was weighed separately. Only the organic fraction was subjected to further analysis.

Water content and total acid number (TAN) of the organic phase were measured via a Mettler Toledo Excellence Titration T5, employing Karl Fischer titration (ASTM E203) and titration methods (ASTM D664), respectively.

The composition of the organic phase was analyzed using a GC/MS instrument (Agilent 7890 A/Agilent 5975 C) equipped with a DB-1701 column (30 m × 0.25 mm × 0.25 μm). Samples were prepared in a 50:50 (v/v) solution of dichloromethane (DCM) and methanol. The MS scanning range was set at *m*/*z* 45–500, with a scan rate of 1.64 scans per s. The MS source and quadrupole temperatures were maintained at 230 and 150 °C, respectively. Samples were injected into the GC at 280 °C (split ratio of 5:1), with helium as the carrier gas (1 mL/min flow rate). The temperature program started with a 5-minute hold at 35 °C, followed by a ramp-up of 3 °C/min to 250 °C. Compound identification was performed manually using the NIST-11 library, and results were presented as normalized GC/MS peak areas to facilitate comparison across different samples. Elemental analysis (CHN) of the organic phase was performed by Eurofins Environmental Testing Sweden AB using the DIN 51732:2014 method.

#### 2.5.2 Gas Analysis

The noncondensable vapors were passed through a gas washing setup to capture as many vapors as possible that had not condensed in the initial condensation stages. The gas composition was then analyzed using an Agilent 490 micro GC, calibrated to detect and measure H_2_, O_2_, N_2_, CO, CH_4_, CO_2_, C_2_H_4_, and C_2_H_6_. The gas analysis also facilitated the estimation of the total gas mass produced during the process. Normalizing the nitrogen flow based on the micro GC readings, the flow rates of other compounds were calculated. The masses of these detected compounds were then determined by converting volume to mass using the ideal gas law, assuming room temperature and atmospheric pressure conditions.

#### 2.5.3 Solid Residue Analysis

The ultimate, proximate, and element analyses of pyrolysis char and coke were carried out by Eurofins Environmental Testing Sweden AB. The ash and moisture contents of pyrolysis char were measured using the standard method (SS-EN ISO 1171:2018, SS-EN ISO 589:2018). Moreover, element analysis was performed using SFS-EN 13656:2020 and SFS-EN ISO 11885:2009 methods.

#### 2.5.4 Br Analysis

In order to collect gaseous bromide in the noncondensable gases, two scrubbing bottles filled with NaOH solution (80 mL, 1 mol/L) were placed after the cooling system to absorb HBr. A gas washing bottle filled with deionized water was placed before the gas measurement to wash the impurities in the gas. Once the reactor and test products reached steady-state conditions, the noncondensable gases were stored in a gas bag for sampling. The sampling period was lasted for 30 min. Bromine analysis was conducted by ALS Scandinavia AB, in accordance with the SS-EN ISO 10 304-1:2009 standard. Moreover, the bromine content in the solid residue and oil was also measured by the external laboratory Eurofins Environmental Testing Sweden AB, using the DIN EN 15408:2011, SS-EN 15408:2011, and SS-EN ISO 17294-2:2016 standardized method.

## 3 Results and Discussion

### 3.1 Batch Pyrolysis in an Ex Situ Catalytic Reactor

A batch scale ex situ catalytic pyrolysis over a HZSM-5 catalyst was conducted to compare the results from a continuous pyrolysis process and investigate the decomposition behavior of the WECB. Thermal pyrolysis was also performed to conduct a comparative analysis with catalytic pyrolysis. Figure 3a displays the product yields resulting from noncatalytic and catalytic pyrolysis of WECB in a batch type reactor. The yield of the solid residue was similar in both cases due to the independent pyrolysis configuration and the same pyrolysis parameters. Noncatalytic pyrolysis predominantly produced oil compared to gas, which yielded 18.56 and 3.14 wt %, respectively. In contrast, using HZSM-5 significantly reduced the yield of organic fraction to 12.67 wt % while increasing the gas yield to 7.32 wt %, verifying the role of HZSM-5 on enhancing cracking reactions and promoting lighter hydrocarbons production. The use of HZSM-5 significantly enhanced the production of aromatic hydrocarbons, such as benzene, toluene, and xylene (BTX), while reducing the oxygenated compounds.^27^ Due to the strong acidity and microporous structure, zeolite catalysts are able to promote cracking, deoxygenation, and aromatization reactions. This investigation highlights the advantages of HZSM-5 in improving the quality and selectivity of pyrolysis oil, demonstrating its potential for sustainable waste conversion and high-value chemical production. The elemental composition of oil from batch pyrolysis is shown in Table S1. Moreover, the detected compounds in the oil were dominated by phenols, while HZSM-5 significantly enhanced the yield of valuable light aromatics, increasing from 8.83 peak area % in the noncatalytic case to 16.08 peak area % under catalytic conditions, while simultaneously reducing oxygenated compounds and heavy organic residues, as presented in Supporting Information in Table S2. Overall, the HZSM-5 catalyst enhanced the efficiency and selectivity of catalytic pyrolysis compared to noncatalytic processes for the WECB.^28^

**Figure 3:**
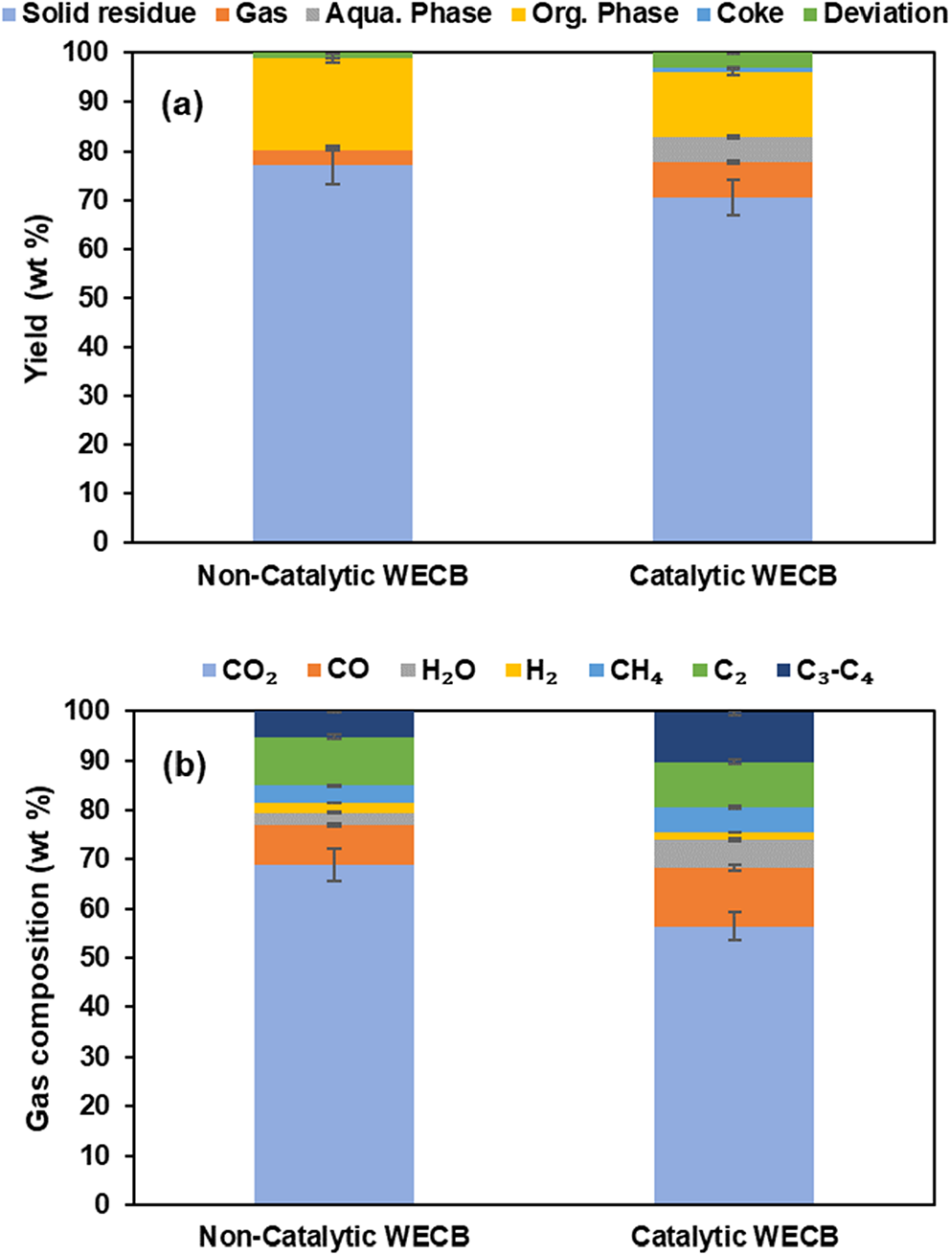
(a) Product yield (b) and gas composition for the batch pyrolysis test.

Figure 3b shows the gas composition from noncatalytic and catalytic pyrolysis of WECB. The results showed that noncatalytic pyrolysis exhibited a high CO_2_ proportion, indicating incomplete deoxygenation.^29^ Catalytic pyrolysis demonstrated an increased yield of CO and light hydrocarbons such as CH_4_, and C_2_–C_4_, emphasizing the catalytic role of HZSM-5 on promoting deoxygenation and hydrocarbon formation.^30^

### 3.2 Continuous Pyrolysis in an Auger Reactor Followed by Catalytic Reactor

#### 3.2.1 Mass Balance

The effect of WHSV was investigated at a pyrolysis temperature of 500 °C and a catalytic temperature of 450 °C. Catalytic pyrolysis was conducted using two WHSV by adjusting the WECB feed rate while keeping a constant catalyst load for vapor upgrading. Figure 4 shows the yields of the pyrolysis products. For the noncatalytic pyrolysis, the liquid yield was 18.0 wt % and the gas yield was 2.7 wt %, respectively, which is consistent with the results from batch pyrolysis. HZSM-5 and CaO, used in a mixed mode, served as a dual catalyst to promote the cracking reaction of pyro-vapors and enhance the bromine reduction and decarbonization of the process. Two different values of WHSV were investigated in this study, namely 0.6 and 1.0 h^–1^. Comparing the product distribution in the presence and absence of a catalyst (HZSM-5/CaO). As can be seen, the gas yield was increased when the catalyst was employed, and a significant reduction of the organic phase was observed. Moreover, the gas yield was 6.5 wt % at WHSV of 0.6 and was down to 6.2 wt % at WHSV of 1.0. The total liquid yield reached a minimum of 12.5 wt % at a WHSV of 0.6 and increased to 16.4 wt % when the WHSV was raised to 1.0. This result indicated that using a catalyst in the catalyst reforming process could significantly promote cracking reactions and produce lighter hydrocarbons. The lowest organic yield was obtained at a WHSV of 0.6, which can be attributed to the relatively higher catalyst content per WECB feeding. The longer residence time of pyro-vapor passing through the catalyst bed leads to more secondary reactions, such as cracking, dehydration, and decarboxylation, resulting in higher gas yields and reduced liquid yields.^31^ The recovery rate of oil (OR, Oil recovery rate = *L*_yield_/volatiles)^4^ followed the same trend and increased from 30.6 to 40.1 wt % as WHSV increased from 0.6 to 1.0. On the contrary, the recovery rate of gas (GR, Gas recovery rate = *G*_yield_/volatiles)^4^ had a minor decrease as WHSV increased. The yields of solid residue (char) in the pyrolysis reactor were similar in all cases, which was 70 wt %, with a 1–2% error due to the nonhomogeneous nature of the feedstock.

**Figure 4:**
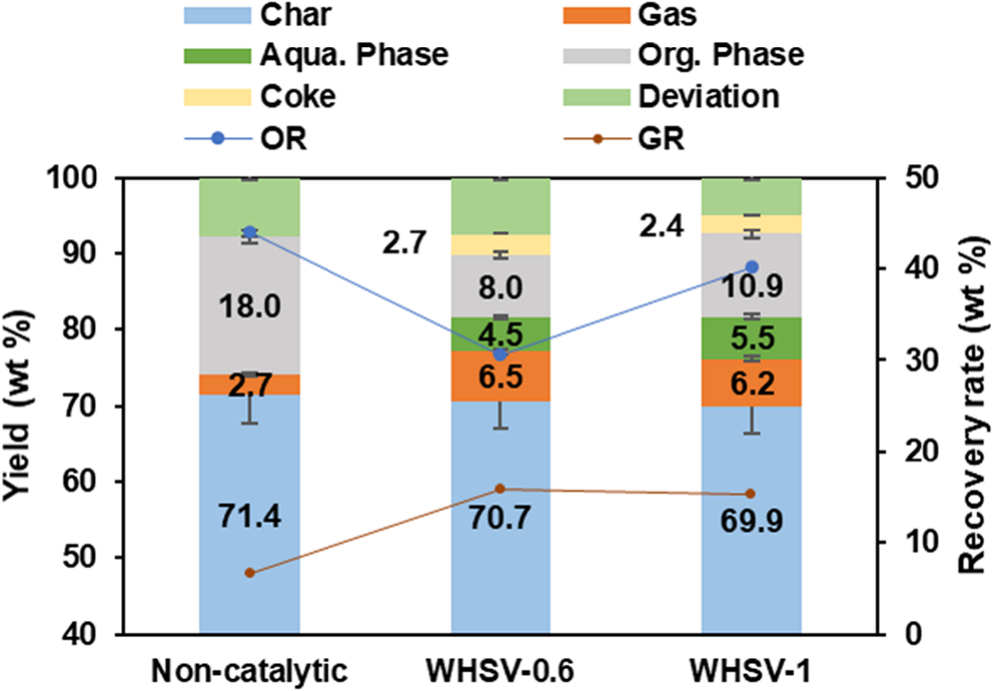
Products yield and recovery ratio of pyrolysis products (oil recovery rate (OR), gas recovery rate (GR)).

#### 3.2.2 Gas Composition

The effect of different WHSV on the gas composition was also investigated, and the results are compared to the result from a noncatalytic run, as shown in Figure 5. For thermal pyrolysis without using the catalyst, CO_2_, CH_4_, and C_2_–C_3_ are the dominant gas components, accounting for 43.2, 16.7, and 25.2 mol %, respectively. The relatively high concentration of CO_2_ correlates with the conversion of carboxylic acids via decarboxylation reactions for the thermal degradation of the WECB.^32^

**Figure 5:**
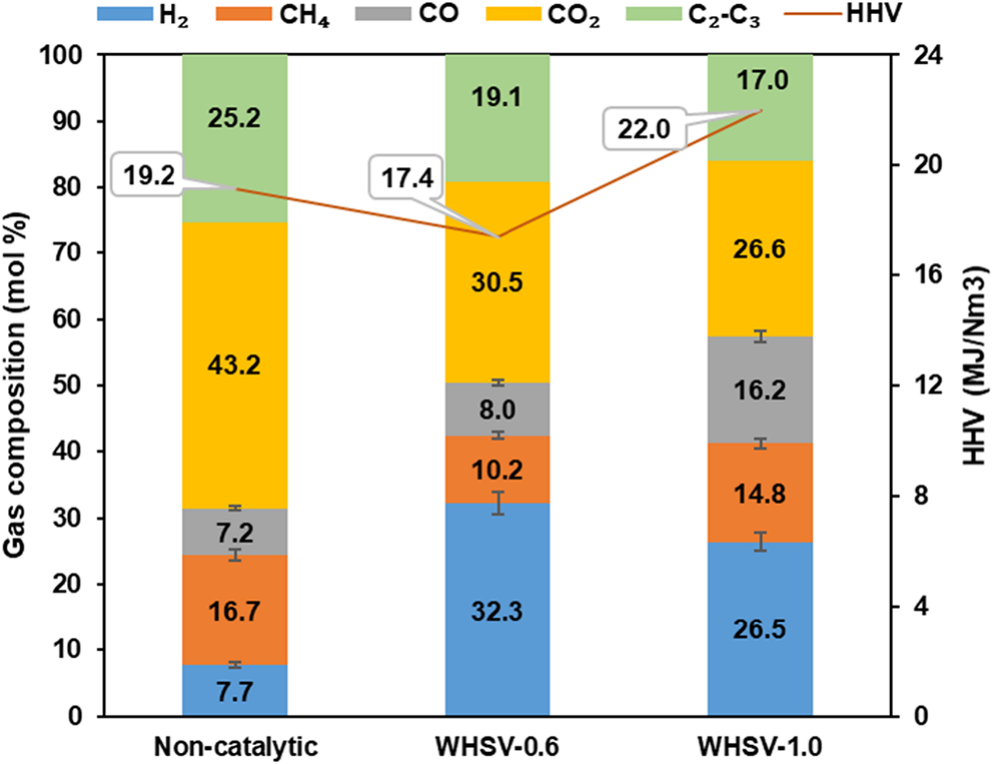
Gas composition in mol % and higher heating value (HHV) of the noncondensable vapors.

The use of the dual-catalyst significantly increased the proportion of H_2_ and CO, while a lower WHSV led to higher concentrations of H_2_. Specifically, at a WHSV of 0.6, the H_2_ concentration was 32.3 mol % compared to 26.5 mol % at a WHSV of 1.0. Furthermore, the concentration of CH_4_, and CO_2_, was reduced at the lower WHSV, suggesting that HZSM-5/CaO, when used as a reforming catalyst, can significantly enhance the cracking of volatile compounds. A lower WHSV (0.6), which corresponds to a longer residence time, promotes the further cracking of small molecular compounds, such as methane and C_2_–C_3_ into hydrogen, resulting in high-quality syngas.^33^ Additionally, the use of a CaO additive was found to significantly reduce the formation of CO_2_ at lower WHSV, while simultaneously enhancing the relative concentrations of H_2_. This observation is consistent with studies suggesting that CaO actively captures CO_2_ by forming CaCO_3_, which promotes the water–gas shift reaction (WGSR) and methane steam reforming, leading to increased H_2_ production.^34^ Furthermore, the formation of coke observed in this process further promotes CO_2_ consumption by the Boudouard reaction, which enhances the effect of CaO in reducing CO_2_ levels and increasing H_2_ yield. This dual role of CaO, both as a decarbonization agent (CO_2_ capture) and as a catalyst for hydrogen-producing reactions together with a cracking catalyst, highlights its effectiveness in optimizing gas composition. However, the prolonged residence time associated with a lower WHSV of 0.6 also reduces the 17.4% decline in the Higher Heating Value (HHV). This reduction is primarily attributed to a diminished concentration of light hydrocarbons, particularly methane and C_2_–C_3_ hydrocarbons, which have significant contributions to the energy content of the produced gas.

HBr and CH_3_Br are the primary bromine-containing gaseous products generated during WECB pyrolysis.^35^ HBr formation occurs as a result of the interaction between bromine free radicals and hydrogen, driven by the increased concentration of hydrogen radicals in the gas phase.^36^ The production of CH_3_Br predominantly originates from the breakdown of methoxy groups attached to phenolic compounds.^35^ Furthermore, a substantial release of CH_3_Br is attributed to the secondary cracking of bromine-containing organic compounds present in the pyrolysis oil, facilitating their transformation to gaseous products. This secondary cracking process highlights the role of pyrolysis conditions in influencing the releasing dynamics of Br-containing volatiles. The use of a catalyst significantly reduced the formation of HBr compared to that of CH_3_Br. This difference could be attributed to the stronger adsorption capacities of CaO for HBr compared to CH_3_Br, making HBr easier to be captured and removed during the process and it could be neutralized due to the alkaline feature of CaO.^35^

#### 3.2.3 C–H–O–Br Distribution

Figure 6 shows the distribution of C, H, O, and Br in different pyrolysis products in all three cases. The elemental yield is calculated based on the weight percentage of the initial element in the feedstock and normalized to 100%. For the carbon element, in all cases, a very small amount of carbon remains in the solid residue and the highest amount can be seen in the liquid phase. Thermal pyrolysis was conducted in an auger reactor at a temperature of 500 °C, resulting in a consistent yield of solid residue across all cases. Consequently, the carbon content in the solid residue yield remained unchanged. In the absence of a catalyst, 56 wt % of the initial carbon was found in the liquid phase. Notably, when the catalyst is used, the carbon content in the gas phase was substantially decreased while the carbon content in the organic phase was increased, reaching approximately 78 wt % at a WHSV of 0.6 and 77 wt % at a WHSV of 1.0. Both the O and H contents displayed a higher percentage in the aqueous phase compared to the organic phase in the catalytic cases. The Br distribution in the pyrolysis products from noncatalytic and catalytic pyrolysis of WECB is illustrated in Figure 6d. Around 44 wt % of the initial Br remained in the solid residue for all of the cases due to the consistent pyrolysis conditions. The second highest Br content was found in the gas phase, followed by that in the liquid phase. For noncatalytic pyrolysis, 38.3 wt % of the initial bromine was detected in the gas and 17.1 wt % in the oil phase. The addition of the catalyst at two different WHSVs resulted in a large decrease in the bromine content of the organic liquid. It might be attributed to the function of the dual-catalyst HZSM-5/CaO in enhancing secondary reactions between Br-containing substances in the feedstock and pyro-vapors, thereby promoting the formation of organobromine compounds in the liquid phase. Additionally, at a lower WHSV of 0.6, a significant reduction of bromine content in the organic liquid was observed, reaching 10.9 wt %. This reduction might be due to the prolonged contact time of pyro-vapors with the catalyst surface caused by the longer residence time, thus promoting the further decomposition of small molecular compounds and the secondary cross-linking reactions facilitated by the acidic catalyst, resulting in the most effective debromination performance.

**Figure 6:**
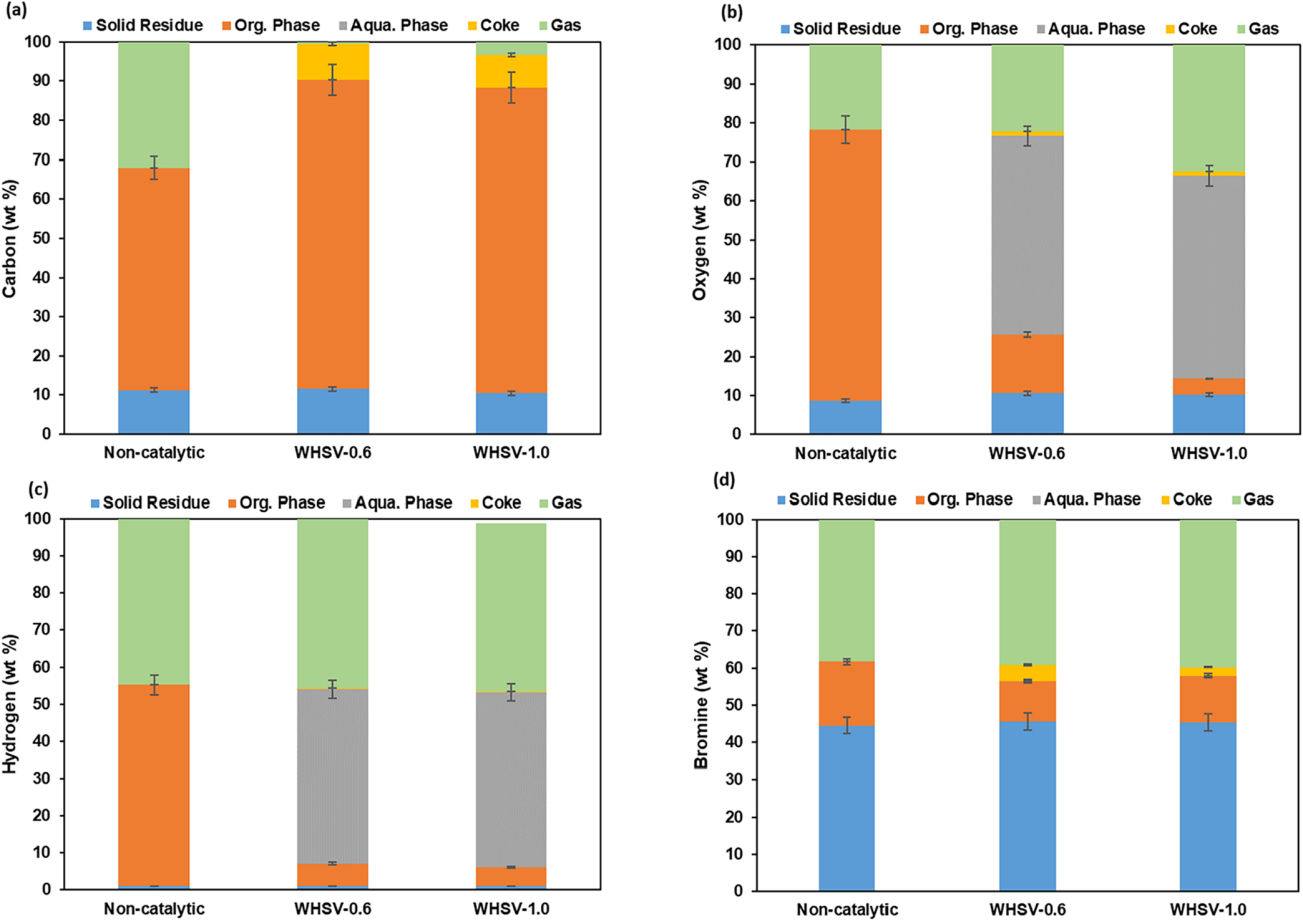
C–H–O and Br transformation over solid residue, oil, coke, and gas products. (a) Carbon, (b) oxygen, (c) hydrogen, and (d) bromine.

#### 3.2.4 Liquid Analysis

The liquid product (oil) was analyzed based on the oil’s elemental composition, bromine distribution, and organic composition. The liquid phase was separated in catalytic cases into an aqueous and an organic phase, and the composition of each phase was analyzed by GC-MS using the same method for all tested cases. The main composition of oil and its peak area percentages are summarized in Table 4. In the noncatalytic pyrolysis, the primary substances in oil include phenol, acids, esters, ketones, benzene, nitriles, and furans. The percentage of phenols in the oil from noncatalytic pyrolysis was 58.7 peak area %, which is mainly derived from bisphenol A-type epoxy resin. This composition is attributed to the relatively low pyrolysis temperature below 550 °C.^35^ Under the use of the dual-catalyst HZSM-5/CaO, phenols were primarily concentrated in the aqueous phase, while monoaromatic hydrocarbons such as benzene, toluene, xylene (BTX), and polycyclic aromatic hydrocarbons (PAHs) were concentrated in the organic phase. The percentage of phenols in organic phase at WHSV of 0.6 decreased from 58.69 to 27.58 peak area %, while the PAH content was increased from 1.04 to 21.60 peak area %. The percentages of BTX were increased to 5.85, 3.45, and 2.68 peak area %, respectively, in the organic phase at a WHSV of 0.6, attributed to secondary cracking facilitated by the strong acidic sites of HZSM-5. Moreover, the yield of acidic compounds decreased, indicating the deacidification performance of CaO.

**Table 4:** Composition of Pyrolysis Oil (GC-MS Peak Area %)

	non-cat	0.6-aqua	0.6-org	1.0-aqua	1.0-org
acid	3.54	0.52	0.62	9.12	0.58
aldehyde	0.51	0.00	0.24	0.00	0.00
esters	2.79	0.00	0.86	0.00	0.08
ether	0.38	0.00	0.21	0.00	0.00
ketone	1.94	1.29	0.27	0.00	1.88
aromatic HC	1.33	0.00	12.60	2.91	3.50
heterocyclic aromatic HC	0.74	0.00	1.55	0.00	1.17
benzene	2.87	0.00	5.85	0.77	7.07
toluene	1.37	0.00	3.45	1.65	2.00
xylene	1.07	0.00	2.68	0.51	1.37
styrene	1.58	0.00	0.43	0.61	5.08
guaiacol	0.05	0.00	0.00	0.00	0.04
phenol	58.69	95.96	27.58	77.32	47.97
cyclic HC	0.00	0.00	2.47	0.00	0.14
PAHs	1.04	0.00	21.60	0.28	10.28
nitriles	3.60	1.85	9.20	3.68	5.63
furan	4.61	0.00	6.78	2.17	6.42
N-containing compounds	2.48	0.00	0.22	0.00	1.97
S-containing compounds	0.88	0.00	0.14	0.00	0.11
Cl-containing compounds	2.03	0.37	0.07	0.00	1.36
Br-containing compounds	2.04	0.00	0.66	0.00	1.96
F-containing compounds	2.77	0.00	2.31	0.98	1.36
P-containing compounds	1.56	0.00	0.11	0.00	0.07
CN-containing compounds	1.69	0.00	0.00	0.00	0.00
Si-containing compounds	0.44	0.00	0.00	0.00	0.66

Reaction pathways for the basic catalytic process can be divided into four steps. First, a neutralization reaction with CaO, which converts acids into (RCOO)_2_Ca and water. Second, there is a decomposition of (RCOO)_2_Ca into ketone and CaCO_3_. Meanwhile, acidic compounds (R-COOH) were thermally cleaved in the presence of CaO and converted to hydrocarbons and CO_2_ via cracking reactions. Furthermore, CO_2_ produced in the reaction may also react with the CaO to form CaCO_3_.^37^

The Weight Hourly Space Velocity (WHSV), defined as the weight of feedstock processed per unit weight of catalyst per hour, notably affects aromatics production during pyrolysis.^17^ A lower WHSV allows for extended contact time between pyro-vapors and catalyst, leading to more extensive catalytic cracking and reforming reactions, which are essential for converting large hydrocarbons and oxygenates into small hydrocarbons like BTX, heterocyclic aromatics, and PAHs. Moreover, the prolonged interaction time with the catalyst at low WHSV enhanced secondary reactions and catalytic performance, such as deoxygenation and hydrogen transfer, further refining intermediate products such as phenols into aromatic hydrocarbons. However, the change in the selectivity of BTX was not significant between WHSV of 0.6 and 1, corresponding to 11.98 and 10.44 peak area %, respectively. Furthermore, excessive residence time due to low WHSV can increase the risk of coke deposition on the catalyst, leading to deactivation of the catalyst and the need for more frequent regeneration processes.^38^ Halogenated species, particularly bromine (Br), in the oil can lead to operational challenges, such as equipment corrosion, which poses significant risks when implementing the process commercially. Therefore, it is important to design a process incorporating a catalyst capable of reducing halogenated compounds in the oil, ensuring improved efficiency, and minimizing the risk of equipment corrosion.

The dual-catalyst HZSM-5/CaO showed a significant reduction of bromine in oil, as illustrated in Table 4. Compared to noncatalytic pyrolysis, the use of HZSM-5/CaO catalysts reduced the bromine concentration in the organic phase from 2.06 to 0.66 peak area % at a WHSV of 0.6, indicating the occurrence of a strong synergistic effect between HZSM-5 and CaO.^39^ The debromination performance of the HZSM-5 catalyst can be evaluated based on the composition of the pyrolysis oil, characterization of the spent catalyst, and the adsorption of volatile brominated compounds, which contributed to bromine removal from the pyrolysis oil.^40^ In addition, the high acidity and porosity of the zeolite catalyst could enhance its debromination performance.

However, the adsorption of Br-containing volatiles was hindered by the capture of CO_2_ in the presence of CaO catalyst during pyrolysis, as reported by Xie et al.^41^ At temperatures below 500 °C, CO_2_ capture led to the formation of CaCO_3_, which contributed to catalyst deactivation by reducing the availability of active sites. To restore the catalytic activity, decomposition of CaCO_3_ into CaO and CO_2_ at higher temperatures is required, which limits the catalytic performance of the dual-catalyst. This decomposition regenerates CaO and creates additional open pores and adsorption sites, improving the diffusion, adsorption, and secondary reactions of Br-containing volatiles.^42^

The results of the Br species distribution in oil detected by GC-MS are presented in Figure 7. The main organic Br-containing compounds in oil are mainly brominated phenols such as phenol 2,6-dibromo, phenol, 2-bromo, phenol, 3-bromo, and brominated aromatic HCs such as benzene 1-bromo-2-methyl, benzene 1-bromo-3-methyl-, benzene 1-bromo-4-ethyl-, benzene 1-(bromomethyl)-2-methyl, benzene 1,4-dibromo-2-methyl, benzene 1,4-dibromo-2-methyl-, benzene bromo, benzene 2-bromo-1,3-dimethyl, benzenamine 4-bromo-2,6-dimethyl-, benzene bromo, benzene 2-bromo-1,3-dimethyl, 1,2-dibromobenzocyclobutene, and ethanone 2-bromo-1-phenyl. Brominated phenols were primarily derived from the breakdown of brominated bisphenol A within the polymer chain, attributed to the cleavage of the isopropylidene bridge and Ph–Br bonds. Among these, 2-bromophenol was the most abundant compound, with its concentration increasing from 0 to 0.66 peak area % as the WHSV was from 0.6 to 1 h^–1^. A comparable trend was observed for 2,6-dibromophenol. At lower WHSV values, extended residence time promoted the secondary cracking of the Ph–Br bond. It facilitated the direct cyclization of brominated phenolic compounds, leading to the production of benzofuran in the pyrolysis oil.^11,43^ Additionally, the active sites supplied by CaO and HZSM-5 enhanced polymer cracking, forming small molecular groups. This process further enabled free radical rearrangements between small molecular radicals and volatile organic radicals, driving reactions that resulted in the production of ethanone, 2-bromo-1-phenyl.

**Figure 7:**
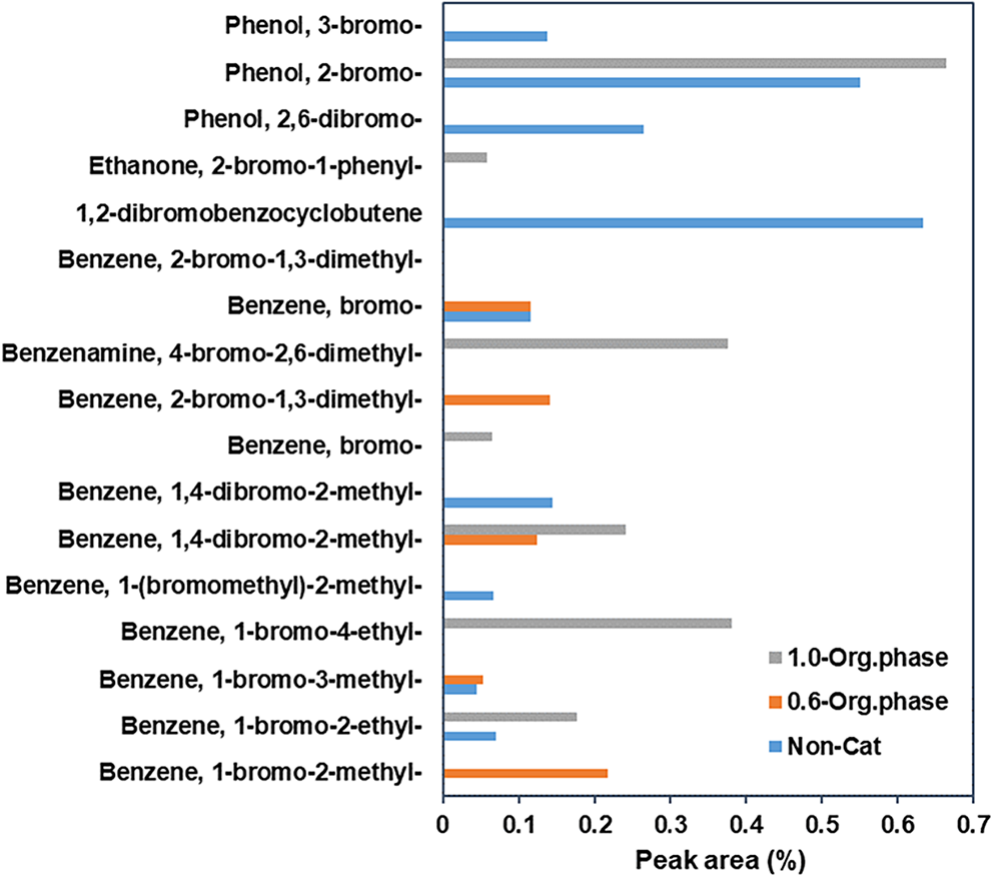
Br species distribution in organic liquid presented as peak area % by GC-MS.

#### 3.2.5 Solid Residue Analysis

The studied WECBs had a relatively low bromine concentration (1.5 wt %), primarily in the form of cross-linked brominated epoxy resin. The solid residue has been analyzed for ultimate, proximate, and mineral analyses, and the results are summarized in Table 5. From the C–H–O–Br distribution analysis and elemental analysis of solid residue, 44 wt % of initial Br remained in the pyrolysis residue. During the pyrolysis of WECB, most bromine is volatilized into the liquid and gas phases as organic and inorganic brominated compounds, and the remaining fraction is retained in the solid residue. This may result from the interaction of bromine free radicals with alkali or alkaline earth metals. Bromine-containing volatiles released during pyrolysis can be retained in the pyrolysis residue through secondary interactions with metals or metal oxides, such as Ca, Mg, and K of the feedstock, primarily associated with metal oxides rather than as free inorganic bromine.^44^ At a pyrolysis temperature of 500 °C, the cleavage of the C–Br bond occurs as an initial step in the decomposition of brominated compounds present in the feedstock.^4^ Moreover, bromine in the solid residue remains resistant to heating due to its binding with the mineral compounds of the feedstock, making it less prone to volatilization during pyrolysis.

**Table 5:** Solid Residue Analysis

proximate analysis (wt %, db)	
	result
moisture content	2 ± 0.2
ash content	86.3 ± 8.6
volatile matter	11.7 ± 0.5
heating value (MJ/kg)	7.1

ultimate analysis (wt %, db)	
	result
C	10.4 ± 0.52
H	1 ± 0.13
N	0.38 ± 0.11
O	0.1

element analysis (mg/kg, db)	
bromine	9500
aluminum	71,000
antimony	2900
arsenic	14
barium	3600
beryllium	0.5
lead	3500
boron	4000
phosphorus	1200
iron	12,000
cadmium	4.9
calcium	68,000
potassium	2400
silicone	100,000
cobalt	13
copper	200,000
chromium	290
mercury	0.08
magnesium	11,000
manganese	1300
molybdenum	11
sodium	1900
nickel	780
tin	12,000
titanium	2000
vanadium	19
zinc	19,000

#### 3.2.6 Br Transformation Mechanism

The predominant brominated flame retardants in WECBs include tetrabromobisphenol A (TBBPA), decabromodiphenyl ether (DDO), decabromodiphenyl ethane (DDE), and 1,2,5,6,9,10-hexabromocyclodecane (HBCD).^45^ These polybrominated compounds are thermally unstable and decompose under pyrolytic conditions, releasing Br^•^ radicals that serve as key flame-retardant agents by interrupting radical chain reactions and suppressing fire propagation.^23^ The presence of Sb_2_O_3_ further accelerates halogen liberation by promoting the formation of volatile antimony halides and oxyhalides during thermal degradation.^46^ As pyrolysis progresses with increasing WHSV, the more bromine content transitions into liquid and gaseous products.^47^ In this study, tetrabromobisphenol A (TBBPA) is identified as the most likely primary brominated flame retardant in WECB.^23^ As the feedstock enters the constant temperature zone of the pyrolysis reactor, the phenyl–Br bond undergoes preferential cleavage, releasing bromine radicals. These Br^+^ radicals then react with hydrogen radicals and small molecular fragments, facilitating the formation of hydrogen bromide (HBr) and organobromine compounds, which are subsequently transferred into the pyrolysis oil and gas phase.^22^ Additionally, a minor fraction of Br can link with carbon, forming C–Br bonds, which remain fixed in the solid residue, as discussed in Section 3.2.5.

According to previous studies conducted by Gao et al. and Liu et al.,^22,48^ a potential mechanism for bromine (Br) transformation during WECB pyrolysis has been proposed based on GC-MS, C–H–O–Br distribution, and gas analyses, as shown in Figure 8. During the catalytic pyrolysis process, bromine-containing organic compounds predominantly release HBr as a primary brominated byproduct. A strong base, calcium oxide (CaO), readily reacts with HBr, neutralizing the acidic gas and forming calcium bromide (CaBr_2_). This transformation process can be categorized into three main stages:

(1) Neutralization reactions: At approximately 450 °C, CaO reacts with HBr via a neutralization reaction, forming CaBr_2_, as described in eq 1.
1 $$\mathrm{CaO}+2\mathrm{HBr}\to {\mathrm{CaBr}}_{2}+H_{2}O$$
  The resulting CaBr_2_ species create lattice defects in CaO, act as active sites that preferentially adsorb organic bromides due to their high surface energy. Moreover, CaO reacts with HBr, potentially improving the adsorption efficiency of organic bromides.^22^
(2) Debromination reactions: The interaction between brominated phenols in oil and calcium species resulted in the debromination of oil. According to molecular orbital theory, the bromine atoms in phenol, referred to as Ph–Br, act as electron donors, providing lone pairs of electrons. These electrons can enter the unoccupied orbitals of calcium ions, forming coordination bonds, as represented in eq 2.^49,50^
2 $$\mathrm{Ph}-\mathrm{Br}+{\mathrm{Ca}}^{2+}/{\mathrm{Ca}}_{\mathrm{atom}}\to \left[\mathrm{Ph}-\mathrm{Br}\cdot \cdot \cdot {\mathrm{Ca}}^{2+}\right]\;or\;\left[\mathrm{Ph}-\mathrm{Br}\cdot \cdot \cdot {\mathrm{Ca}}_{\mathrm{atom}}\right]$$
  This structural weakening suggests that the C–Br bond is more prone to breakage when Ca^2+^ is positioned adjacent to the *ortho*- and *para*-bromine atoms of bromophenol.
(3) Regeneration reactions: After debromination, phenol radicals react with hydrogen radicals to form phenol, as reported by Gao et al.^22^ Simultaneously, Ca^2+^ undergoes regeneration through the eq 3.
3 $${\mathrm{Ca}}^{2+}{\mathrm{Br}}^{•}\to {\mathrm{Ca}}^{2+}+{\mathrm{Br}}^{•}$$
  The Br^+^ free radicals subsequently interact with hydrogen free radicals to generate HBr, which CaO effectively captures. This allows Ca^2+^ to continuously extract bromine atoms from bromophenol, thereby maintaining catalytic activity.

**Figure 8:**
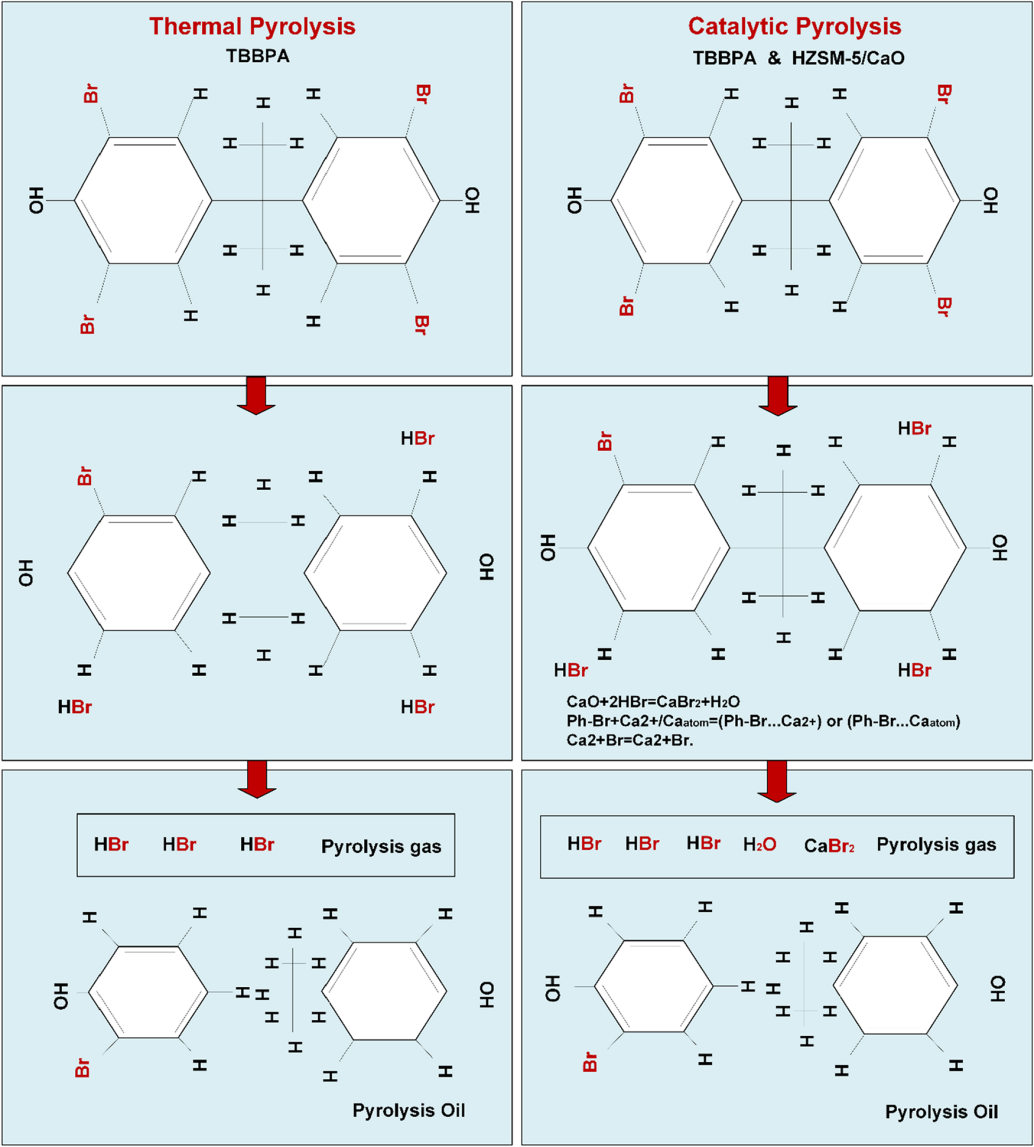
Proposed Br transformation in the catalytic pyrolysis of WECBs in an auger reactor.

## 4 Conclusions

This study systematically investigated the catalytic pyrolysis of Waste Electronic Circuit Boards (WECBs) using a dual-catalyst HZSM-5/CaO in a continuous pyrolysis system. The main findings can be summarized as follows:

1. The batch pyrolysis results showed that HZSM-5 significantly enhanced the yield of valuable light aromatics, increasing from 8.83 peak area % in the noncatalytic case to 16.08 peak area % under catalytic conditions, while simultaneously reducing oxygenated compounds and heavy organic residues.
2. The dual-catalyst HZSM-5/CaO plays a crucial role in improving product quality by facilitating cracking, deoxygenation, and decarbonization reactions. Catalytic pyrolysis resulted in a decrease in liquid yield but an increase in gas yield, promoting the formation of lighter hydrocarbons. The catalytic effect was also evident in the quality of the liquid products. The proportion of phenolic compounds in the organic phase dropped drastically from 58.7 peak area % in noncatalytic pyrolysis to 27.6 peak area % at WHSV 0.6. Simultaneously, the formation of monoaromatic hydrocarbons, such as BTX, as well as PAHs was significantly enhanced. This transformation indicates that the acidic sites of HZSM-5 promoted secondary cracking and aromatization, while CaO contributed by neutralizing acidic compounds and reducing oxygenates, thereby producing higher-value aromatic hydrocarbons suitable as chemical feedstocks.
3. The introduction of HZSM-5/CaO greatly influenced gas composition, particularly in terms of hydrogen enrichment. At WHSV 0.6, the H_2_ concentration rose to 32.3 mol %, compared with 26.5 mol % at WHSV 1.0. In parallel, CO_2_ emissions were significantly suppressed due to the strong CO_2_ capture capacity of CaO, which facilitated reforming reactions such as the water–gas shift and reforming reactions.
4. One of the most critical outcomes was the substantial reduction of bromine in the pyrolysis products. The bromine concentration in oil was reduced from 2.06 wt % under noncatalytic conditions to 0.66 wt % at WHSV 0.6 with the dual-catalyst. In addition, approximately 44 wt % of the initial bromine was immobilized in the solid residue as stable inorganic bromides, primarily bound to mineral components. This effective debromination was attributed to the synergistic action of HZSM-5 and CaO, where CaO neutralized HBr and adsorbed bromine species, while HZSM-5 enhanced the decomposition of brominated organics. Such bromine control is crucial for reducing environmental risks and corrosion problems in industrial-scale pyrolysis systems.

The dual-catalyst HZSM-5/CaO demonstrated its strong ability for reducing organobromine in pyrolysis oil and great potential for industrial-scale pyrolysis applications, providing an efficient route for waste valorization.

## Supplementary Material



## Data Availability

The data underlying this study are available in the published article and its Supporting Information.

## Notes

Experimental plan and product characteristics (PDF)
