# Supplementary material for: Bromine Transformation during Catalytic Pyrolysis of Waste Electronic Circuit Boards (WECBs) in an Auger Reactor over the Dual-Catalyst HZSM-5/CaO
Source: ACS Omega. 2025 Nov 6;10(45):54720–32. doi: 10.1021/acsomega.5c08152 (PMC12631318; doi:10.1021/acsomega.5c08152)
Supplement: Supplementary file 1 [file ao5c08152_si_001.pdf]

Supporting information for

Bromine Transformation During Catalytic Pyrolysis of Waste Electronic Circuit Boards (WECBs) in an Auger Reactor over Dual Catalyst HZSM-5/CaO

Samina Gulshan [, †], Hoda Shafaghat [‡], André Selander [§], Hanmin Yang [*, †], Panagiotis Evangelopoulos [‡], Pär G Jönsson [†] and Weihong Yang [†]*

[†] KTH Royal Institute of Technology, Department of Materials Science and Engineering, Brinellvägen 23, SE-114 28 Stockholm, Sweden

[‡] Division of Bioeconomy and Health, Department of Biorefinery and Energy, RISE Research Institutes of Sweden AB, SE-941 28 Piteå, Sweden

[§] Paper and Forest Product Manufacturing, SCA Munksund AB, SE 941 87 Piteå, Sweden.

***Corresponding author**

Samina Gulshan

Address: Brinellvägen 23, 114 28, Sweden

E-mail: saminag@kth.se

Hanmin Yang

Address: Brinellvägen 23, 114 28, Sweden

E-mail: hanmin@kth.se

Content

Table S1. Elemental composition of oil from batch pyrolysis

Table S2. Composition of pyrolysis oil from batch pyrolysis (GC-MS peak area %)

Figure S1. The calibration curve for the feeding screw of an auger reactor

Figure S2. Pyrolysis oil: catalytic (left) and non-catalytic (right

Table S1. Elemental composition of oil from batch pyrolysis

	Non-catalytic WECB		Catalytic WECB	
**(wt.%)**	Aqueous phase	Organic phase	Aqueous phase	Organic phase
**C**	4.13	67.34	2.33	72.29
**H**	10.5	6.8	10.83	6.52
**N**	0.98	2.09	0.19	2
**S**	0.04	0.03	0.03	0.03
**O**	83.49	22.9	86.01	18.64
**HHV (MJ/kg)**	1.49	28.46	0.90	30.50

Table S2. Composition of pyrolysis oil from batch pyrolysis (GC-MS peak area %)

	**Non-catalytic WECB**		**Catalytic WECB**	
**Compounds type**	Aqueous phase	Organic phase	Aqueous phase	Organic phase
Alcohols	0.84	0.00	0.00	0.00
Acids	4.10	0.00	0.00	0.00
Ethers	0.00	0.00	0.00	0.00
Esters	0.00	0.00	0.00	0.00
Furans	0.61	0.86	0.00	0.40
Aldehydes	0.71	0.00	0.00	0.00
Phenols	83.88	76.48	86.43	69.95
Aromatic oxygenates	0.00	0.00	0.00	0.00
Ketones	4.54	0.00	4.38	0.00
Oxygenates C2-C3	0.00	0.00	0.00	0.00
Other oxygenates	0.00	0.00	0.00	0.00
Paraffins	0.00	0.00	0.00	0.00
Olefins	0.00	0.00	0.00	0.00
Acetylenes	0.00	0.00	0.00	0.00
Aromatic HCs	0.00	0.48	0.00	0.46
Monoaromatic HCs	0.00	1.86	0.00	6.90
Styrenes	0.00	1.22	0.00	0.74
BTX	0.00	5.75	0.00	8.44
Naphthalenes	0.00	0.57	0.00	2.42
PAHs	0.00	0.02	0.00	0.36
Saturated cyclic HCs	0.00	0.00	0.00	0.00
Unsaturated cyclic HCs	0.00	0.01	0.00	0.01
Other HCs	0.00	0.00	0.00	0.00
Nitriles	3.74	9.55	5.96	9.89
N-containing compounds	1.49	1.55	0.15	0.23
Br-containing compounds	0.00	0.29	0.00	0.00
Si-containing compounds	0.00	0.39	0.00	0.02
B-containing compounds	0.00	0.00	0.00	0.00
Cl-containing compounds	0.10	0.00	2.75	0.12
N,Cl-containing compounds	0.00	0.00	0.32	0.00
N,Br-containing compounds	0.00	0.98	0.00	0.00

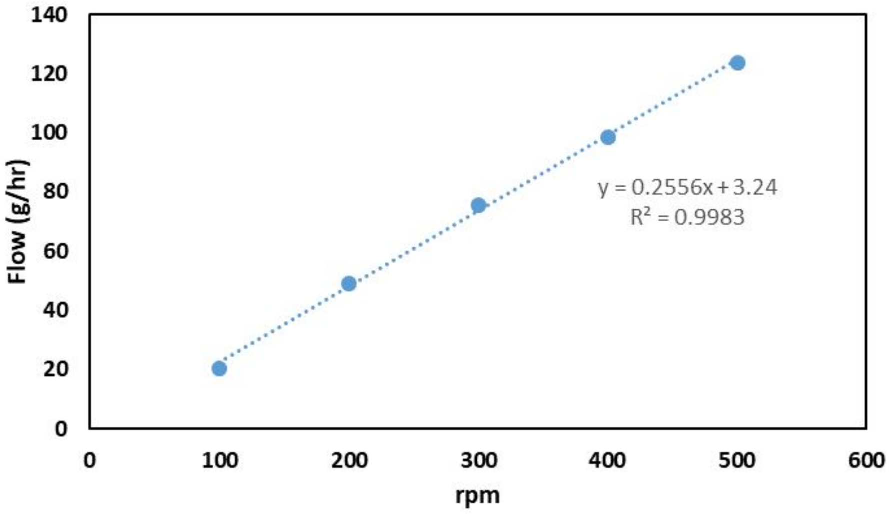

Figure S1. The calibration curve for the feeding screw of an auger reactor

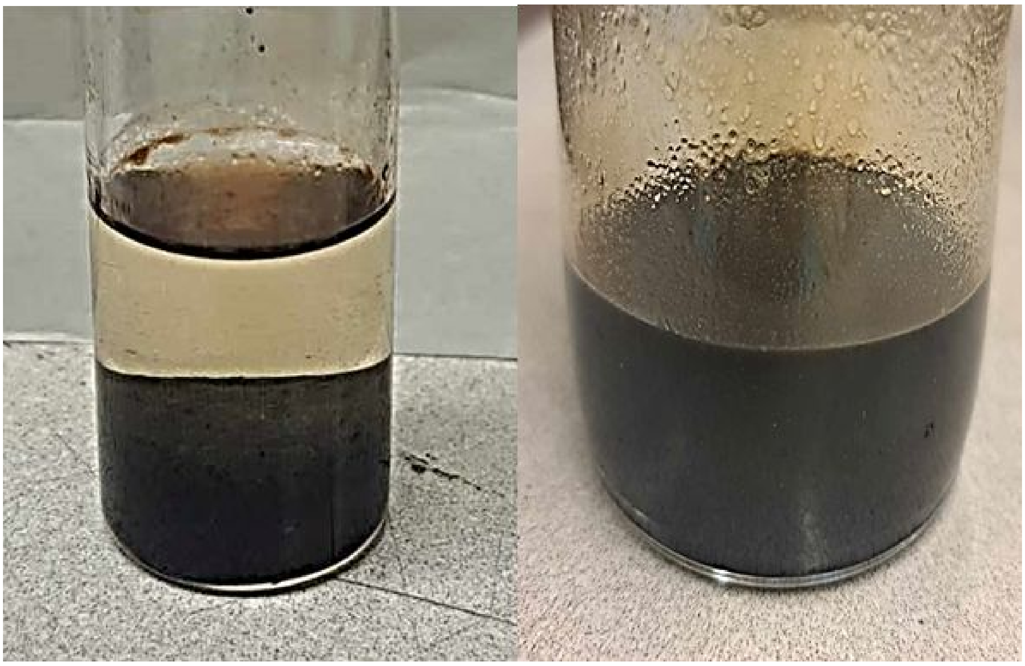

Figure S2. Pyrolysis oil: catalytic (left) and non-catalytic (right)
